# Is health promotion in sports clubs associated with adolescent participants’ fruit and vegetable consumption?

**DOI:** 10.1093/eurpub/ckad158

**Published:** 2023-09-04

**Authors:** Laura Heikkilä, Raija Korpelainen, Tuula Aira, Lauri Alanko, Olli J Heinonen, Sami Kokko, Jari Parkkari, Kai Savonen, Kerttu Toivo, Maarit Valtonen, Tommi Vasankari, Jari Villberg, Marja Vanhala

**Affiliations:** Department of Sports and Exercise Medicine, Oulu Deaconess Institute Foundation sr, Oulu, Finland; Research Unit of Population Health, Faculty of Medicine, University of Oulu, Oulu, Finland; Medical Research Center, Oulu University Hospital and University of Oulu, Oulu, Finland; Department of Sports and Exercise Medicine, Oulu Deaconess Institute Foundation sr, Oulu, Finland; Research Unit of Population Health, Faculty of Medicine, University of Oulu, Oulu, Finland; Medical Research Center, Oulu University Hospital and University of Oulu, Oulu, Finland; Faculty of Sport and Health Sciences, University of Jyväskylä, Jyväskylä, Finland; Sports Medicine Clinic, Foundation for Sports and Exercise Clinic, Helsinki, Finland; Sports and Exercise Medicine Clinic, Central Finland Hospital Nova, Jyväskylä, Finland; Paavo Nurmi Centre & Unit for Physical Activity and Health, University of Turku, Turku, Finland; Faculty of Sport and Health Sciences, University of Jyväskylä, Jyväskylä, Finland; Faculty of Sport and Health Sciences, University of Jyväskylä, Jyväskylä, Finland; Tampere Research Center of Sports Medicine, Tampere, Finland; Kuopio Research Institute of Exercise Medicine, Kuopio, Finland; Department of Clinical Physiology and Nuclear Medicine, Science Service Center, Kuopio University Hospital, Kuopio, Finland; Tampere Research Center of Sports Medicine, Tampere, Finland; UKK Institute of Health Promotion Research, Tampere, Finland; Finnish Institute of High Performance Sports KIHU, Jyväskylä, Finland; UKK Institute of Health Promotion Research, Tampere, Finland; Faculty of Medicine and Health Technology, Tampere University, Tampere, Finland; Faculty of Sport and Health Sciences, University of Jyväskylä, Jyväskylä, Finland; Department of Sports and Exercise Medicine, Oulu Deaconess Institute Foundation sr, Oulu, Finland; Research Unit of Population Health, Faculty of Medicine, University of Oulu, Oulu, Finland; Medical Research Center, Oulu University Hospital and University of Oulu, Oulu, Finland

## Abstract

**Background:**

Although sports clubs’ potential for health promotion is acknowledged, research on whether they promote healthy eating is limited. We aimed to evaluate Finnish youth sports clubs’ health promotion orientation, as well as associations between sports clubs’ health promotion orientation, coaches’ nutritional discussions and sports club participants’ (SPs’) fruit and/or vegetable consumption.

**Methods:**

The cross-sectional study included 554 SPs aged 14–16 years, 275 club officials and 311 coaches. Participants replied to questionnaires about sports clubs’ health promotion and their own health behaviours, including dietary habits. Health promotion orientation was estimated using a Health Promoting Sports Club (HPSC) index (range 0–22) and nutritional discussions and fruit and/or vegetable consumption as frequencies. A mixed-effects multivariable logistic regression was used to analyse the associations.

**Results:**

Most sports clubs (69%) had a high health promotion orientation, but the variation between the clubs was wide (HPSC index range 5–21). SPs’ daily fruit and/or vegetable consumption was associated with female gender [odds ratio (OR) 3.48, 95% confidence interval (CI) 2.23–5.42, *P* < 0.001], better self-rated health (OR 3.26, 95% CI 1.13–9.41, *P* = 0.03), higher average school grades (OR 1.67, 95% CI 1.04–2.67, *P* = 0.03), and SPs’ responses that their coach had often discussed nutrition (OR 2.11, 95% CI 1.41–3.14, *P* < 0.001).

**Conclusion:**

Although sports clubs’ orientation towards health promotion was mostly high, it seems not to be enough to promote healthy eating among adolescent participants. Instead, coaches’ nutritional discussions were associated with adolescents’ fruit and/or vegetable consumption.

## Introduction

Health-promoting dietary habits, including fruit and vegetable consumption and family meals, tend to decrease during adolescence.^1^ The consumption of fruit and vegetables plays a central role in food-based dietary guidelines globally,^2^ although 53% of 15-year-old adolescents eat neither fruit nor vegetables daily.^1^

It is well known that settings are an important approach in health promotion.^3^ A setting for health is the place or social context in which people engage in daily activities in which environmental, organizational and personal factors interact to affect health and wellbeing.^4^ Traditional settings for health promotion are, for example, cities, workplaces and schools, but newer settings, such as sports clubs, have also been recognized.^5^

Youth sports clubs reach approximately half of all children and adolescents worldwide.^6^ Sports clubs represent a potential setting for health promotion given their informal educational nature.^7^ Health-promoting sports clubs focus not only on sports-related objectives but also on health-related policies and practices, a supportive ideology and a safe environment.^8^

Sports clubs’ setting-based health promotion can be illustrated as a three-level model in which acting on the preceding level affects the next level’s activities.^9^ The macro level includes a club’s health promotion orientation and policies, the meso level comprises club officials’ guidance activity towards coaches and the micro level refers to coaches’ health promotion activity towards sports club participants (SPs).^9^

Despite sports clubs’ potential for health promotion, research on how health behaviours and health can be promoted through sports club settings remains limited.^10^ Sports clubs have had a moderate orientation towards health promotion and identified health promotion ideologies, but health promotion principles and activities have been poorly realized in practice.^8^^,^^11^^,^^12^ Most studies have focused on a single health promotion level, such as only micro-level activities, instead of adopting a settings-based approach.^10^^,^^13^ Additionally, little is known about the role of sports clubs in promoting healthy eating.

According to a report among Australian junior soccer teams,^14^ sports clubs’ policies and practices regarding the promotion of healthy eating have been highly variable. Thirty-nine percent of Australian community-level junior sports clubs reported that, during training and competitions, they advise players regarding healthy dietary habits, such as eating fruit and drinking water.^15^ Ninety-one percent of sports club representatives replied that clubs have a responsibility to promote healthy dietary choices, but only 3–8% of sports clubs had a written policy about the promotion of healthy eating.^14^^,^^15^

Although the importance of nutrition for health and performance is undeniable, the relationships between sports clubs’ health promotion and adolescent SPs’ dietary habits have not been studied. This study aimed to (i) describe Finnish youth sports clubs’ health promotion orientation and (ii) examine the associations between sports clubs’ health promotion orientation, coaches’ nutritional discussions and the frequency of adolescent SPs’ fruit and/or vegetable consumption.

## Methods

This cross-sectional study was based on baseline data obtained from the ongoing longitudinal Finnish Health Promoting Sports Club (HPSC) study.^16^ The data were collected in cooperation with the University of Jyväskylä, the UKK Institute and six National Centres of Excellence in Sports and Exercise Medicine in Finland during the 2013–14 period. The study was performed in accordance with the Declaration of Helsinki. Ethical approval was received from the Ethics Committee of the Health Care District of Central Finland (record number 23U/2012). Written informed consent was obtained from all participants.

### Study sample

The study protocol has been described in detail earlier.^16^ In brief, the sampling of the sports clubs was completed in two stages, considering, for example, sports type and main competition season. Data were collected during the main competition season: January–May 2013 for winter sports and August–December 2013 for summer sports. Complementary data were gathered between January and May 2014.

Sports club participants aged 14–16 years were randomly selected from 156 clubs representing the ten most popular sports in Finland: basketball, cross-country skiing, floorball, ice hockey, skating, soccer, gymnastics, orienteering, swimming and track and field. Overall, 759 of 1889 SPs (40%) participated.

In addition to the SPs, 3–5 club officials and 3–5 coaches from each club were invited to take part in the study. The officials were, for example, the chairman of the club or the head of coaching or junior activities. The coaches were included if they were coaching 14- to 16-year-old adolescents at the time. In total, 313 of 625 officials (50%) and 281 of 593 coaches (47%) responded to the study questionnaire.

Two hundred and thirteen study participants (205 SPs, 2 officials and 6 coaches) were removed from the data: (i) who disallowed their answers’ use for research purposes; (ii) who did not fill out the questionnaire adequately; (iii) whose main sport’s club was not involved in the study; (iv) adolescents aged 13 or 17 and (v) adolescents with missing data regarding gender (*n *=* *15), age (*n *=* *67) and/or sports club’s health promotion orientation (*n *=* *27). The study included 554 SPs, 311 officials and 275 coaches from 137 sports clubs. Data collection was conducted using internet-based questionnaires.

### Health promotion

The health promotion orientation of the sports clubs (the macro level) was estimated using the HPSC index (range 0–22).^8^ The index comprises 22 questions based on a five-point Likert scale: (i) does not describe the club at all, (ii) describes the club very little, (iii) describes the club to some extent, (iv) describes the club well and (v) describes the club very well. The officials’ and coaches’ answers were recoded to a two-point scale (0 = describes our club to some extent at the most and 1 = describes our club well or very well) and summed.

The sports clubs were classified into three categories: HPSC index values 0.00–10.99 indicate low, 11.00–14.99 indicate moderate and 15.00–22.00 indicate high health promotion orientation.^8^ To describe various dimensions of sports clubs’ health promotion orientation, four sub-indices were calculated for policies (maximum of eight), ideologies (maximum of two), practices (maximum of seven) and environment (maximum of five).^8^

The items on sports clubs’ health promotion guidance activity and coaches’ health promotion activity (later, nutritional discussions) focused on nutrition in this study. Sports clubs’ health promotion guidance activity towards coaches (the meso level) was asked from the officials and coaches. They replied to a question about how often, during the previous 6 months, officials have instructed coaches to discuss the basics of nutrition with SPs. Regarding coaches’ nutritional discussions with SPs (the micro level), the coaches and SPs answered questions regarding how often, during the last 6 months, coaches have discussed the basics of nutrition with SPs. The answer options were divided into two groups: often (often/very often) and seldom (never/seldom).

### Fruit and/or vegetable consumption

SPs’ fruit and vegetable consumption was estimated with the question: ‘How many times a week do you usually eat the following foods: 1) vegetables and 2) fruits?’ The answer options were divided into two groups: daily (every day, once a day/every day, more than once) and less than daily (5–6 days a week/2–4 days a week/once a week/less than once a week/never). The question was based on the Health Behaviour in School-aged Children (HBSC) study’s food frequency questionnaire, which is valid for ranking children and adolescents according to the consumption of food items.^17^

### Other measures

Body mass index (BMI) was calculated as weight (kg)/height (m^2^) based on the self-reported data. Body size perception and self-rated health were assessed using questions from the HBSC study.^18^ Body size perception was evaluated with the following question:^19^ ‘Do you think your body is … (much too thin/a bit too thin/about the right size/a bit too fat/much too fat)?’ The answer options were divided into two groups: about the right size and others. Self-rated health was queried, and the answer options were categorized as good (excellent/good) or poor (fair/poor).

School grade average was queried using the following question: ‘What was the grade average (all subjects) on your latest report card?’ Eight answer options were dichotomized into good to excellent (8.0–10.0) and adequate to satisfactory (4.0–7.9). Socioeconomic status (SES) was evaluated with the Finnish version of the Family Affluence Scale, Version II.^20^

Experience (years) in the main sport was calculated from the reported starting age in the main sport. Sports disciplines were divided into four sport groups: aesthetic, ball game, endurance and power sports.^21^ Track and field, without further description, was classified as a power sport. Cross-country skiing, skating, gymnastics, orienteering and swimming were considered weight-sensitive sports.^22^

Physical activity was measured in a randomly selected sample of adolescents who participated in preparticipation screening for the HPSC study (*n *=* *308).^16^ The adolescents were asked to use a hip-worn, light tri-axial accelerometer (Hookie AM20 Activity Meter, Hookie Technologies Ltd., Helsinki, Finland) for seven consecutive days, except for during showers and water activities. Swimmers’ (*n *=* *27) accelerometer data were excluded because the accelerometer cannot be used during water activities.

The accelerometer is a valid tool for the continuous monitoring of physical activity among adolescents^23^ and adults.^24^ The device measured and stored tri-axial data in g-units, with a 100 Hz sampling frequency. Raw acceleration data were segmented into 6 s epochs, and mean amplitude deviation (MAD) was calculated for each epoch. These MAD values were converted into metabolic equivalents (METs). Time spent on moderate (3.0–5.9 METs) and vigorous (≥6.0 METs) physical activity was combined to form moderate to vigorous physical activity (MVPA).

Officials’ and coaches’ physical activity, dietary habits and smoking were assessed with questions from large Finnish population-based surveys.^25^^,^^26^ Those who self-reported at least 150 min of moderate-intensity endurance training and/or at least 75 min of vigorous endurance training per week, as well as at least two sessions of muscle strength training per week, were considered to meet the recommendation on physical activity. Coaching experience among coaches and experience in the current position among officials were reported in years. Age, gender, education and alcohol use were also queried.

### Statistical analyses

The normality of the continuous variables was examined. SPs with missing data on daily fruit and/or vegetable consumption (*n *=* *15, 2.2%) and the main independent variables were not included in the analyses. Differences between the groups were tested using cross-tabulation and χ^2^ tests for categorical variables and independent samples *t*-tests or Mann–Whitney *U* tests for continuous variables. The mean HPSC index was calculated for each club, and club-specific averages were used in the analyses.

The associations between sports clubs’ health promotion and the frequency of adolescent SPs’ fruit and/or vegetable consumption were analysed using multilevel mixed-effects logistic regression analysis. Two levels of data hierarchy were considered in the model, and club level acted as a random effect. Gender, school grade average, self-rated health, family SES, SP’s response that his/her coach had often discussed nutrition with them, sports type (individual or team sports) and main competition season (winter or summer sports) were included in the analysis as covariates. Potential interactions were tested for sports type and main competition season, and sports type × main competition season was used as interaction term. A multilevel mixed-effects logistic regression analysis was performed with Stata 17 (StataCorp, USA), and other analyses were performed using SPSS Version 27 (IBM Corporation, USA). A *P* values <0.05 were considered statistically significant.

## Results

### Health promotion

Most of the sports clubs (69%) self-reported a high health promotion orientation, 25% a moderate orientation and 7% a low orientation (table 1). The clubs’ mean HPSC index was 15.61 (SD 3.12, range 5–21). The officials estimated their own club’s health promotion orientation to be higher than the coaches did (*P* < 0.001). Health promotion orientation did not differ between aesthetic, ball game, endurance and power sports (*P* = 0.313).

**Table 1:** Sports clubs’ health promotion orientation, measured via the HPSC index among club officials and coaches

	Clubs (*n *=* *125)^a^	Clubs (*n *=* *120)^a^	Clubs (*n *=* *137)^a^
	Officials	Coaches^b^	Total
HPSC index	16.11 (3.91)	14.92 (3.77)***	15.61 (3.12)
Policy index	5.69 (1.60)	5.30 (1.61)**	5.54 (1.33)
Ideology index	1.86 (0.36)	1.81 (0.41)	1.83 (0.33)
Practice index	4.68 (1.72)	4.20 (1.63)**	4.49 (1.39)
Environment index	3.88 (0.94)	3.60 (0.95)**	3.75 (0.76)
Health promotion orientation, *n* (%)			
Low	9 (7)	15 (13)	9 (7)
Moderate	31 (25)	34 (28)	34 (25)
High	85 (68)	71 (59)	94 (69)
HPSC index by sports discipline groups			
Aesthetic (*n *=* *21)	17.06 (3.58)	15.32 (3.40)	16.29 (3.47)
Ball game (*n *=* *60)	15.69 (4.74)	15.01 (3.93)**	15.27 (3.32)
Endurance (*n *=* *32)	16.33 (2.90)	14.35 (4.67)	15.73 (3.13)
Power (*n *=* *11)	16.62 (1.99)	16.23 (2.75)	16.44 (2.21)

Values are means (SD) unless otherwise stated.

HPSC, Health Promoting Sports Club.

a Officials responded from 125 clubs, coaches from 120 clubs and officials and/or coaches from 137 clubs.

b Differences between the officials (*n *=* *311) and the coaches (*n *=* *275) estimated using Mann–Whitney *U* test:

** *P *<* *0.01,

*** *P *<* *0.001.

Health promotion ideology (i.e. ‘fair play’ and ‘everyone plays’ ideologies) was rated to be the best implemented. The mean environment (a club’s infrastructures and social interactions) and policy (rules and priorities for health promotion) indices also reached the high health promotion level. Daily activities related to health promotion were the least recognized: the mean practice index was classified as moderate.

When the sports clubs were divided into high and low or moderate in terms of health promotion orientation, club officials’ guidance activities towards coaches were more common among highly than lowly or moderately health-promoting sports clubs (*P* < 0.001) (table 2). Half of the officials (51%) responded that some of the club officials have often given instructions to coaches about discussing nutrition, whereas only 28% of the coaches replied that they had received those instructions from officials.

**Table 2:** Characteristics of the sports clubs’ officials (*n *=* *311) and coaches (*n *=* *275) according to clubs’ health promotion orientation

Characteristics	Officials (*n *=* *311)		Coaches (*n *=* *275)		Total (*n *=* *586)	
	LM	H^a^	LM	H^a^	LM	H^a^
	(*n *=* *89)	(*n *=* *222)	(*n *=* *85)	(*n *=* *190)	(*n *=* *174)	(*n *=* *412)
Age (years), mean (SD)	46 (7)	46 (9)	41 (11)	40 (11)	44 (10)	43 (10)
Male, *n* (%)	52 (58)	131 (59)	62 (73)	119 (63)	114 (66)	250 (61)
Experience (years), median (IQR)	3 (2–7)	4 (2–6)	9 (5–13)	7 (4–12)	5 (3–10)	5 (3–10)
Higher education, *n* (%)	46 (52)	109 (49)	35 (41)	92 (48)	81 (47)	201 (49)
Physical activity: recommendation met vs. not^b^, *n* (%)	14 (26)	32 (21)	19 (35)	33 (27)	33 (30)	65 (24)
Vegetables, fruit and/or berries: 6–7 days a week, *n* (%)	50 (56)	125 (56)	42 (49)	111 (58)	92 (53)	236 (57)
Smoking: daily, *n* (%)	2 (2)	11 (5)	3 (4)	16 (8)	5 (3)	27 (7)
Alcohol use: weekly, *n* (%)	40 (45)	81 (37)	33 (39)	55 (29)	73 (42)	136 (33)*
Health promotion guidance activity						
Officials have often instructed coaches to discuss nutrition with SPs, *n* (%)	26 (29)	131 (59)***	13 (15)	63 (33)**	39 (22)	194 (47)***
Nutritional discussions						
I have often discussed nutrition with SPs, *n* (%)			61 (72)	141 (74)		

H, high health promotion orientation; LM, low to moderate health promotion orientation; SP, sports club participant.

a Differences between the LM and the H sports clubs estimated using cross-tabulation and χ^2^ test, independent samples *t*-test or Mann–Whitney *U* test:

* *P* < 0.05,

** *P* < 0.01,

*** *P* < 0.001.

Sports clubs’ health promotion orientation was measured using the Health Promoting Sports Club index.

b Numbers do not match due to missing values (*n *=* *385).

Regarding coaches’ health promotion activity, 74% of the coaches reported that they had often discussed nutrition with SPs, and 49% of the SPs replied that their coach had often discussed nutrition with them. Coaches’ responses regarding nutritional discussions with SPs did not differ between highly and lowly or moderately health-promoting sports clubs (*P* = 0.768).

### Fruit and/or vegetable consumption

Of the SPs, 57% were males, and 52% consumed fruit and/or vegetables daily (table 3). The SPs who consumed fruits and/or vegetables daily were more often female (*P* < 0.001) and more likely to be members of families with higher SES (*P* = 0.023) than those who did not eat fruit and/or vegetables daily. No differences were found in age, BMI or accelerometer-measured MVPA between the daily fruit and/or vegetable consumption groups.

**Table 3:** Characteristics of the adolescent sports club participants (*n *=* *554) according to fruit and/or vegetable consumption

Characteristics	Male (*n *=* *313)		Female (*n *=* *241)		Total (*n *=* *554)	
	Less	Daily^a^	Less	Daily^a^	Less	Daily^a^
	(*n *=* *193)	(*n *=* *120)	(*n *=* *75)	(*n *=* *166)	(*n *=* *268)	(*n *=* *286)
Male, *n* (%)					193 (72)	120 (42)***
Age (years), mean (SD)	15.0 (0.5)	15.1 (0.6)	14.9 (0.7)	15.0 (0.6)	15.0 (0.6)	15.0 (0.6)
BMI (kg/m^2^), mean (SD)	20.7 (2.3)	21.3 (2.1)*	21.0 (2.3)	20.7 (2.2)	20.8 (2.3)	21.0 (2.1)
Accelerometer-measured MVPA (min/day)^b^, mean (SD)	98 (31)	105 (36)	79 (23)	79 (27)	91 (30)	89 (33)
Experience in main sport (years), mean (SD)	7.2 (2.8)	7.7 (2.5)	6.8 (3.0)	6.6 (3.0)	7.1 (2.9)	7.0 (2.8)
Individual sports, *n* (%)	43 (22)	31 (26)	27 (36)	88 (53)*	70 (26)	119 (42)***
Summer sports, *n* (%)	56 (29)	43 (36)	34 (45)	99 (60)	90 (34)	142 (50)***
Weight-sensitive sports, *n* (%)	36 (19)	23 (19)	45 (60)	97 (58)	81 (30)	120 (42)**
Body size satisfaction, *n* (%)	139 (72)	91 (76)	41 (55)	115 (69)*	180 (67)	206 (72)
Self-rated good health, *n* (%)	189 (98)	117 (98)	65 (87)	162 (98)**	254 (95)	279 (98)
Good school grade average, *n* (%)	116 (60)	90 (75)**	65 (87)	150 (91)	181 (68)	240 (84)***
SES^c^, mean (SD)	6.5 (1.5)	6.7 (1.5)	6.2 (1.6)	6.7 (1.6)*	6.4 (1.6)	6.7 (1.5)*
Club’s HPSC index, mean (SD)	15.9 (2.9)	15.9 (3.1)	16.1 (2.9)	16.2 (2.8)	16.0 (2.9)	16.1 (2.9)
My coach has often discussed nutrition with me, *n* (%)	83 (43)	72 (60)**	28 (37)	87 (52)*	111 (41)	159 (56)**

BMI, body mass index; HPSC, Health Promoting Sports Club; MVPA, moderate to vigorous physical activity; SES, socioeconomic status.

a Differences between those who ate fruit and/or vegetables daily and less than daily estimated using cross-tabulation and χ^2^ test or independent samples *t*-test:

* *P* < 0.05,

** *P* < 0.01.

b From randomly selected sample of adolescents: 133 males and 148 females.

c Estimated using the Family Affluence Scale, Version II,^20^ and higher values indicate higher SES.

In a multilevel mixed-effects logistic regression analysis, SPs’ daily fruit and/or vegetable consumption was more frequent among girls than among boys, and it was positively associated with SPs’ responses regarding their coaches’ nutritional discussions, school grade average and self-rated health (table 4). Sports type and main competition season had significant interaction with fruit and/or vegetable consumption: SPs from team sports with a main competition season in winter had lower odds of daily fruit and/or vegetable consumption than SPs from individual summer sports. Those team winter sports were ice hockey (37%), floorball (32%), basketball (20%) and synchronized skating (12%). Family SES was not independently associated with SPs’ daily fruit and/or vegetable consumption (*P* = 0.068).

**Table 4:** OR, 95% CI and *P*-values for factors associated with daily fruit and/or vegetable consumption among sports club participants (*n *=* *554)

Variable	OR	95% CI	*P*-value
Gender			
Female	3.48	2.23–5.42	<0.001
Male	1		
My coach has discussed nutrition with me			
Often	2.11	1.41–3.14	<0.001
Seldom or never	1		
Self-rated health			
Good	3.26	1.13–9.41	0.029
Poor	1		
School grade average			
Good to excellent	1.67	1.04–2.67	0.034
Adequate to satisfactory	1		
Sports type × main competition season			
Team × winter sports	0.48	0.27–0.85	0.012
Team × summer sports	0.75	0.39–1.45	0.395
Individual × winter sports	0.61	0.29–1.29	0.191
Individual × summer sports	1		

Associations were analysed using multilevel mixed-effects logistic regression adjusted for socioeconomic status.

## Discussion

This study investigated Finnish youth sports clubs’ health promotion orientation and the associations between sports clubs’ health promotion orientation, nutritional discussions and sports club participants’ fruit and/or vegetable consumption. The sports clubs self-reported a high orientation towards health promotion in this study. However, responses about health promotion guidance activity and nutritional discussions varied between the officials, coaches and adolescent participants. The SPs who reported that their coaches had often discussed nutrition with them were more likely to eat vegetables and/or fruits daily, but sports clubs’ health promotion orientation and coaches’ responses about their nutritional discussions were not associated with SPs’ daily fruit and/or vegetable consumption.

Two-thirds of the youth sports clubs reported a high health promotion orientation in this study. Similarly to the previous studies,^8^^,^^11^^,^^27^ health promotion ideology and environment were better recognized, whereas the policy and practice sub-indices had lower values. The officials estimated their club’s health promotion orientation to be better than the coaches did, yet the answers varied between the clubs. Sports clubs’ health promotion orientation appeared have improved as compared with Finnish data collected 6 years earlier,^8^ in which only one-quarter of the clubs had a high health promotion orientation. One previous study^27^ also found that the largest proportion of the French sports clubs’ coaches rated their club as highly health promoting, albeit with a large amount of variability between the clubs. Additionally, health promotion orientation has been moderate among Irish Gaelic Athletic Association (GAA) sports clubs, including hurling and Gaelic football,^11^ or even low among Flemish youth sports clubs representing diverse sports disciplines.^12^

Sports clubs’ comprehensive health promotion orientation creates a higher level of guidance activity aimed at coaches.^28^ We also found that club officials’ guidance activities towards coaches were more common in highly health promoting sports clubs than in lowly to moderately health-promoting sports clubs. However, coaches’ discussions about nutrition with SPs did not differ according to a club’s health promotion orientation. Perceptions of health promotion guidance activity and nutritional discussions differed between the officials, coaches and adolescent SPs. This result confirms previous research findings indicating that sports clubs’ health promotion is not comprehensively recognized, and activities aimed at young participants are ineffective.^29^^,^^30^

Although overall health promotion orientation at the macro level was mostly high, it seemed not to be reflected in the sports clubs’ daily activities of healthy eating promotion. A study among French sports clubs has similarly found that coaches considered health promotion to be a relevant aim for sports clubs but health promotion practices were weakly implemented.^27^ High variation in nutrition education policies and practices has also been reported among Australian sports clubs with junior soccer teams^14^: all the clubs reported that they recommend fruit or water be provided to players, but only 8% of the clubs had a written healthy eating policy.

SPs who replied that their coach had often discussed nutrition with them were more likely to eat fruit and/or vegetables daily. This finding suggests that coaches’ and SPs’ discussions about nutrition would have beneficial effects on adolescent SPs’ fruit and/or vegetable use. However, coaches’ responses to nutritional discussions and sports clubs’ health promotion orientation were not associated with SPs’ fruit and/or vegetable consumption. Adolescents who reported daily fruit and/or vegetable consumption may be more inclined to receive guidance on healthy eating than other youths. Similarly, we previously found that coaches’ discussions about substance use, as a health promotion activity, were associated with a higher prevalence of substance use among SPs,^31^ suggesting that sports clubs’ health promotion activities are more reactive than preventive.

Sports type and main competition season were included as an interaction term in the regression model. Adolescents who participated in team sports with the main competition season in the winter had lower odds of daily fruit and/or vegetable consumption than those in individual summer sports. SPs in team winter sports were mainly ice hockey, floorball and basketball players. Previous studies concerning main competition season and sports types are scant. One previous study^32^ found that participation in team sports was a strong risk factor for increased fast food intake among adolescent boys and potentially due to sports participation, they do not have enough time for meals at home. In the present study, SPs replied to the questionnaire in the middle of the main competition season, and due to seasonal variation in fruit and vegetable consumption,^33^ daily fruit and/or vegetable consumption may have been higher in summer sports than in winter sports.

Adolescent SPs’ dietary habits are also related to many other, non-sports-related factors. Family background has been reported to be one determinant of adolescents’ fruit and vegetable consumption.^34^ However, SES was not independently associated with adolescent SPs’ fruit and/or vegetable consumption in this study. Instead, female gender, school grade average and self-rated health were positively associated with adolescents’ daily fruit and/or vegetable consumption. These findings are in accordance with our previous study conducted among adolescent SPs and non-participants,^35^ as well as other earlier studies among adolescents.^34^^,^^36^^,^^37^

This study, for the first time, investigated youth sports clubs’ healthy eating promotion using a multilevel approach: both clubs’ orientation for health promotion, officials’ guidance activity towards coaches, and coaches’ discussions about nutrition were considered. The main strength of this study is a representative sample of adolescent SPs from different regions and the 10 most popular sport disciplines in Finland, as well as their clubs’ officials and coaches. A relatively large sample enables a comparison between sport types and genders, as well as the results can be generalized to Finnish youth sports clubs. Multilevel modelling used in this study allows possible clustering inside separate sports clubs.

Some limitations of our study must be considered. First, the cross-sectional design does not allow any conclusions about causality. Second, 60% of the invited SPs and 51% of the invited officials and coaches did not participate in the study, which may have resulted in sampling bias. Third, the use of self-reported data may have led to social desirability bias and the overestimation of sports clubs’ health promotion orientation and activities. Fourth, we have no further information about coaches’ nutritional discussions, and these may have been related to nutritional factors other than fruit and vegetable consumption, such as sufficient water or protein intake. Finally, only fruit and/or vegetable consumption was used as a measure of dietary habits. However, increasing fruit and vegetable consumption appears to improve overall diet quality^38^ and is associated with adolescents’ health, such as mental health and a reduced risk of metabolic syndrome.^39^^,^^40^

In conclusion, sports clubs’ general orientation towards health promotion was mostly high, but health promotion policies and practices were less commonly implemented. Perceptions about the coaches’ nutritional discussions varied between the officials, coaches and adolescent participants. Sports club participants’ responses that their coaches have often discussed nutrition, school grade average, self-rated health and female gender were positively associated with their daily fruit and/or vegetable consumption. The results complement previous research indicating that although promoting a healthy lifestyle is an important goal for sports clubs, health promotion activities aimed at adolescents are ineffective. Future studies should examine which policies and practices are effective in sports clubs’ healthy eating promotion and how they can be implemented in sports clubs’ daily activities.

## Data Availability

The data underlying this article cannot be shared publicly due to the privacy of individuals that participated in the study. The data will be shared on reasonable request to the corresponding author.
